# Complete response to alectinib in ALK-fusion metastatic salivary ductal carcinoma

**DOI:** 10.1038/s41698-023-00378-9

**Published:** 2023-04-11

**Authors:** Jacob J. Adashek, Surendra Sapkota, Rodrigo de Castro Luna, Tanguy Y. Seiwert

**Affiliations:** 1 Department of Oncology, The Sidney Kimmel Comprehensive Cancer Center, The Johns Hopkins Hospital, Baltimore, MD USA; 2 Department of Internal Medicine, Saint Agnes Hospital, Baltimore, MD USA; 3 Department of Radiology and Radiological Science, The Johns Hopkins Hospital, Baltimore, MD USA

**Keywords:** Head and neck cancer, Tumour biomarkers, Molecular medicine

## Abstract

The advent of next-generation sequencing (NGS) has allowed for the identification of novel therapeutic targets for patients with uncommon cancers. It is well known that fusion translocations are potent driver of cancer pathogenesis and can render tumors exquisitely sensitive to matching targeted therapies. Here we describe a patient with ALK-fusion positive widely metastatic salivary ductal carcinoma, who achieved a durable complete response from alectinib, a potent and specific ALK tyrosine kinase inhibitor. This case serves as another reminder that ALK-fusions can be targeted regardless of histology and can afford patients dramatic and durable benefit. It also emphasizes the need for insurance coverage for such beneficial therapies. While ALK fusions are exceedingly rare in salivary ductal carcinoma, the presence of multiple other targetable aberrations supports the recommendation for universal NGS testing for such tumors.

## Introduction

Salivary ductal carcinoma (SDC) is a rare and aggressive cancer found mostly in the parotid salivary gland in older patients. It is associated with a poor prognosis, often characterized by rapid progression, early metastatic spread, and a high rate of disease recurrence after surgery and adjuvant therapy^1^. The treatment of metastatic SDC (mSDC) often includes a platinum-based chemotherapy regimen (i.e. doxorubicin and cisplatin) with modest benefit^2^. The 5‐year and 10‐year survival rates for mSDC are 43.5% and 14.5%, respectively^3^. It is widely known that mSDC can express androgen receptor (AR) and harbor HER2 amplifications mimicking features of prostate and breast cancer. Hence both AR and HER2 staining by immunohistochemistry is often considered. The responses to HER2 based therapies have been reported at 60–70% and to AR blocking agents between 18 and 53%^2^. Furthermore PD-L1 expression is variable, but durable benefit has been described with immune checkpoint inhibition^4^.

Next-generation sequencing (NGS) has allowed for a more comprehensive identification of novel therapeutic targets which can lead to improved outcomes for patients with such oncogene-driven cancers^5^. This coupled with the continued rapid development of targeted therapeutics can help to get the right drugs to the right patient even for rare cancers^6–8^. Fusion events in particular are oncogenic drivers of carcinogenesis and ideal targets with dramatic responses^9^.

There has been a paucity of case reports that describe various ALK rearrangements in patients with mSDC. Various studies have found SDC to harbor ALK rearrangements in 4–6% of cases; a rare event in a rare tumor^10,11^. Despite this finding, there have not been any reports of using an ALK-directed therapeutic strategy to treat these patients.

Tissue agnostic approvals have allowed for patients to receive therapies regardless of the histology of origin and have led to significant clinical benefit. Targeting ALK-fusions in other histologies aside from non-small cell lung cancer has been shown to derive both radiographic response and clinical benefit^12–14^. The n-of-1 approach to customize therapies based on patients’ NGS reports is likely paramount to improving outcomes in metastatic cancers^15^. This case represents employment of such an approach with a favorable outcome for a patient with mSDC.

## Results

### Case presentation

**Fig. 1:**
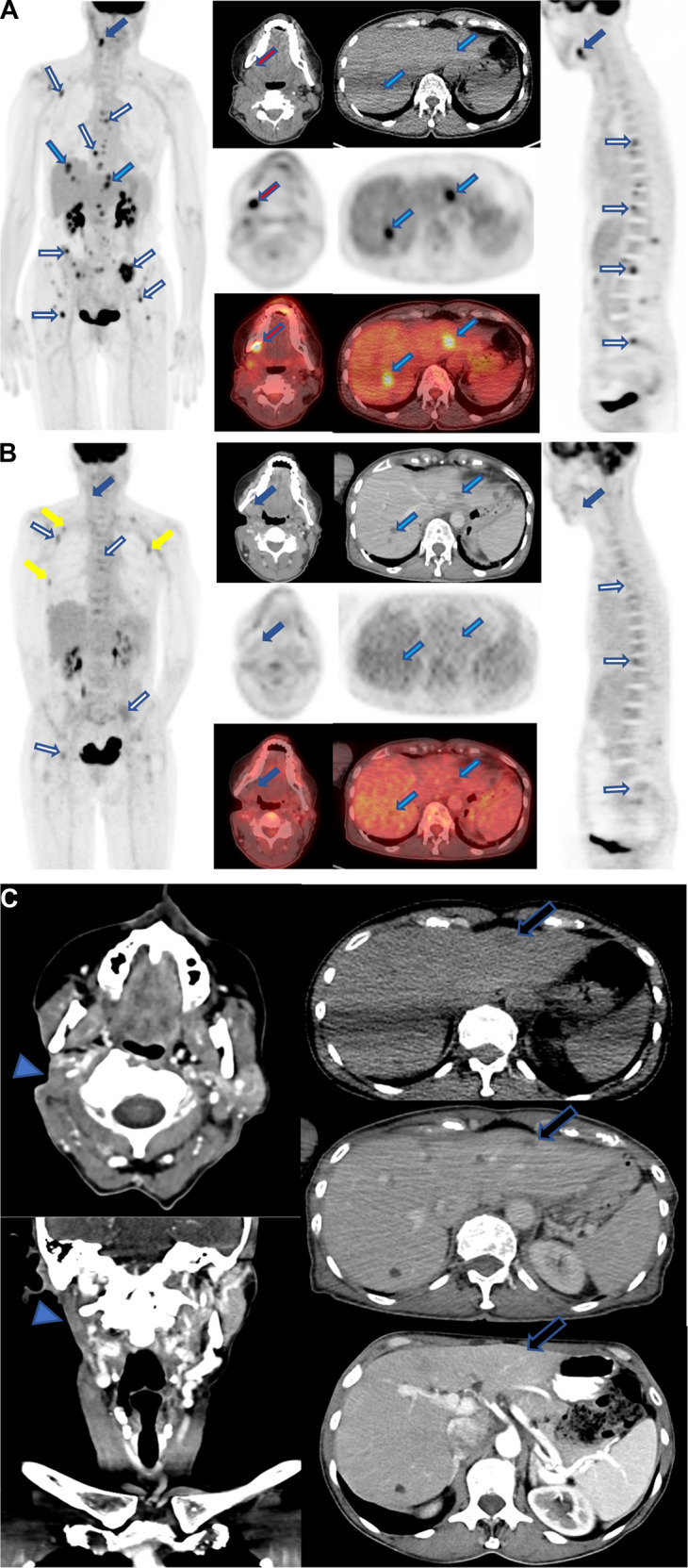
PET/CT Imaging showing evolution of tumor burden before treatment and while on alectinib. **A** Pre-treatment 18 F-FDG PET-CT scan (from right to left; 3D whole-body MIP PET, axial contrast-enhanced CT, PET, and PET/CT fused images at the neck and liver levels, and sagittal PET image) showing markedly FDG-avid right neck level 1B metastasis with SUV max of 4.6 (green arrows), liver metastases with SUV max of 4.8 (blue arrows) and osseous metastases with SUV max of 7.2 (black arrows). **B** 18 F-FDG PET-CT scan at 1 month on treatment (from right to left; 3D whole-body MIP PET, axial contrast-enhanced CT, PET, and PET/CT fused images at the neck and liver levels, and sagittal PET image) showing complete resolution of right level 1B hypermetabolic metastasis (green arrows), complete resolution of the abnormal metabolism of previously seen multiple hypermetabolic liver nodules, and reduced FDG activity in all previously seen hypermetabolic osseous metastases (black arrows). Note that there is new focal FDG activity in the bilateral ribs, correlating with no healed fractures (yellow arrows). **C** Contrast-enhanced CT axial and coronal images (left column) 3 months on treatment revealing status post right parotidectomy with no residual malignancy in the surgical bed (arrowhead) and no nodal disease; axial CT images (right column; from top to bottom: pre-treatment, and at 1/3 month on treatment) evidencing reduction (pre-treatment: 1.6 cm; at 1 month on treatment:1.0 cm), and complete resolution of segment 2 liver metastasis on 3 month-posttreatment CT (white arrows).

A 65-year-old woman initially noted swelling of the right parotid. Initial workup included cross-sectional imaging with a computed tomography (CT) scan which did not reveal a discrete lesion; however, noted thickening of the parotid tail. Swelling progressively worsened and 4 months later she underwent an ultrasound-guided biopsy, which revealed a 2 cm mass. Pathology was reported as a salivary gland neoplasm of uncertain malignant potential and again observation was chosen. However, the neck mass continued to grow, and a follow up neck CT was notable for an infiltrative lesion in the right parotid gland measuring 4.2 × 1.7 cm with possible metastatic nodes in the adjacent right neck measuring up to 12 mm. The patient underwent a right parotidectomy with ipsilateral neck dissection. The surgical pathology revealed a high-grade, poorly differentiated carcinoma diffusely infiltrating the salivary gland with extra-glandular soft tissue extension and perineural invasion. There were 24 positive lymph nodes in the 37 examined with the largest lymph node measuring 2 cm with extra-nodal extension. Immunohistochemical stains demonstrated that tumor cells were positive for CK7, GATA3, and androgen receptors, but negative for HER2, CD117, ER, PR, p53, p63, and SOX10 with a Ki-67 index of approximately 50%. She underwent a positron emission tomography (PET) scan after surgery, which showed an additional hypermetabolic right level 1B lymph node consistent with metastasis, and numerous hypermetabolic lesions in both hepatic lobes consistent with both locoregional and distant metastases. Of note numerous osteolytic and CT occult hypermetabolic lesions were noted and consistent with widespread osseous metastases including involving bilateral ribs, cervical, thoracic, lumbar, and sacral vertebra, bilateral pelvis, and left femoral neck (Fig. 1A). A CLIA-certified laboratory, Strata Oncology, NGS and RNA sequencing was performed on the resected specimen and revealed an EML4-ALK fusion (breakpoints: coordinate chr2:42491871; chr2:29446394).

The patient was started on alectinib 600 mg twice daily for which she paid herself due to denial by the insurance company despite multiple appeals. The patient had severe osseous pain spending the majority of her days in bed at the time of initiation of alectinib.

Within two weeks of starting alectinib she had marked improvement in symptoms. A PET scan performed after one month of alectinib therapy compared to pre-treatment showed a complete resolution of the hypermetabolic lymph node at right level 1B previously seen and complete resolution of FDG avid focus within the right level 2A, complete resolution of abnormal metabolism of all multiple hypermetabolic liver nodules, no new hepatic hypermetabolic lesions with FDG above liver background (Fig. 1B).

A CT scan of her neck, chest, abdomen, and pelvis performed at three months on therapy revealed complete resolution of liver metastasis and no new sites of metastasis or lymphadenopathy (Fig. 1C). She has tolerated alectinib 600 mg twice daily for over eight months with no reportable adverse events and no evidence of disease progression or recurrence. A repeat CT of the chest, abdomen, and pelvis performed at eight months confirmed ongoing complete response without any signs of progression. The patient provided verbal informed consent, which was recorded in her chart, for her imaging and case to be published.

## Discussion

This case highlights multiple salient topics in the evolving landscape of oncology therapeutics. It recognizes the tumor agnostic potential of ALK rearrangements in cancer, which have shown to respond to ALK-directed therapies outside of non-small cell lung cancer^12–14^. It also highlights the importance of access to such life-saving therapies with insurance companies serving as arbiters and in the absence of NCCN guidelines or FDA approvals may lead to poor outcomes.

Large basket trials including all solid tumors with ALK-rearrangements are likely necessary to confirm this concept similar to RET fusions and the efficacy of RET-directed therapies^16–18^. An issue in doing this is the rarity of ALK-rearrangements found in about 0.2% in cancers, which highlights the challenge to provide adequate data to the FDA and insurers^19^.

The response rates to fusions have been shown to be drastic for different cancers and may also depend on the fusion partners (in this case, EML4-ALK is the classic NSCLC reported translocation). In this case as seen in NSCLC the superiority of alectinib compared to an earlier generation ALK inhibitor, such as crizotinib, the authors wanted to avoid potential resistance that can occur with crizotinib, but that alectinib can overcome as well as the lower rates of toxicity. Without an attempt to target such alterations, it is hard to predict response and furthermore may render a tumor resistant to other therapies^9^. The dramatic response that our patient experienced after receiving alectinib highlights the potential for a dramatic “Lazarus effect” with prolonged benefit.

This brings up the question of ‘right to try’ for patients with metastatic cancers as well as the importance of compassionate use programs from pharmaceutical companies, which often help^20^. Given the rarity of ALK translocations in salivary duct cancers there will not be any trial or FDA approval short of a tumor agnostic approval, which the FDA has recently approved for other oncogenic aberrations (i.e., *BRAF V600E*, *NTRK*, *RET*, among others)^21^. The approval and insurance coverage process is problematic for rare tumor types with rare targetable alterations, and such patient undoubtedly oftentimes fare poorly in our current health care system^22^. Despite this, broad genomic profiling using a large panel for patients with mSDC may provide insight into novel therapeutics as it did for our patient.

Our patient continues to have clinical benefit with an ongoing complete response eight months after therapy initiation, which highlights the importance of precision medicine and routine molecular profiling in such cases to address some of the inequities apparent for rare tumors^5,6,9,15,20,23–26^. The authors report a case of a patient with ALK-fusion mSDC receiving alectinib and achieving a complete response; additional studies are warranted in this patient population.

## Methods

A 65-year-old woman underwent a CT for facial swelling with a biopsy that confirmed SDC with immunohistochemical stains which were positive for CK7, GATA3, and androgen receptor with a Ki67 index of approximately 50%, and negative for SOX10, p63, CD117, estrogen receptor, progesterone receptor, and p53. She also had a PET-CT scan that showed metastatic disease and her tissue was sent to Strata Oncology (https://strataoncology.com/), a CLIA-certified laboratory, where NGS and RNA sequencing (https://strataoncology.com/wp-content/uploads/2022/12/Gene_List_SO-SPEC-003v7.pdf) was performed on the resected specimen, which identified an EML4-ALK fusion. The patient was started on an ALK inhibitor, alectinib, and had complete resolution of her cancer which is ongoing at eight months.

### Reporting summary

Further information on research design is available in the Nature Research Reporting Summary linked to this article.

## Supplementary information

REPORTING SUMMARY

## Data Availability

Data sharing not applicable to this article as no datasets were generated or analysed during the current study.

## Code availability

Code sharing not applicable to this article as no codes were generated or analysed during the current study.
